# Diffuse planar xanthomas on the bilateral breasts

**DOI:** 10.1016/j.jdcr.2023.08.004

**Published:** 2023-08-19

**Authors:** Zoha K. Momin, Swapneel J. Patel, Katherine Gordon

**Affiliations:** Department of Dermatology, University of Texas Southwestern Medical Center, Dallas, Texas

**Keywords:** breast xanthomas, diffuse planar xanthoma, normolipidemic, skin of color

## Introduction

Xanthomas are localized deposits of lipid-rich histiocytes (also known as foam cells) in the connective tissue. Various subtypes of xanthomas are described in the literature, including eruptive, tuberous, tendinous, planar, and verrucous.^1^ Cutaneous xanthomatous eruptions can be associated with systemic diseases such as metabolic disease, familial hyperlipidemias, and lymphoproliferative disorders.^1^^,^^2^

First described by Altman and Winklemann in 1962, diffuse planar xanthomas (DPXs) present as asymptomatic, asymmetric plaques involving the periorbital, neck, upper portion of the trunk, and/or flexural regions.^3^^,^^4^ Affected patients are normolipidemic, with an increased incidence of underlying hematologic malignancies. Although DPX has been reported across groups of patients with different age, sex, and body site, to our knowledge, here we present the first case of biopsy-confirmed DPX isolated to the bilateral breasts.

## Case report

A 47-year-old African American woman presented to our academic center with thin, atrophic plaques on her bilateral upper breasts. These skin findings had been present and stable for 4 years. She denied any associated pain or pruritus or other body sites with similar findings. Moreover, she denied any recent breast trauma, masses, pain, swelling, or nipple discharge or retraction. She avoided underwire bras and topical medications on her breasts. She had a medical history of ovarian granulosa theca cell tumor with subsequent excision and a strong family history of breast cancer. The patient was undergoing mammography and breast magnetic resonance imaging every alternate 6 months given her family history of premenopausal breast cancer. Her most recent mammogram was obtained 6 months before with benign findings. She was referred to the dermatology department by the breast oncology department for further evaluation of these lesions.

**Fig 1:**
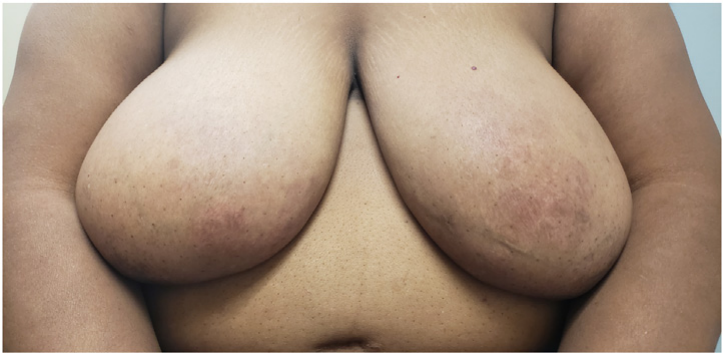
Frontal view of the bilateral breasts.

**Fig 2:**
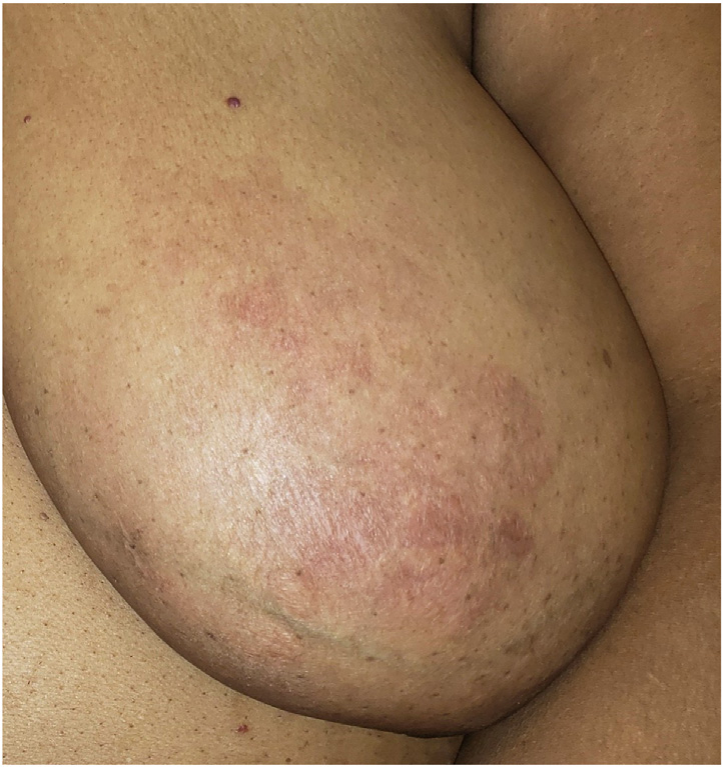
Frontal view of the left breast.

Physical examination revealed thin, atrophic, annular pink plaques overlying the bilateral breasts, sparing the nipple and areola (Figs 1 and 2). No breast masses or nipple discharge were noted. Further laboratory studies included an unremarkable lipid profile (well-controlled on statin therapy), serum-free light chains with mild changes in her free kappa light chains, and minor elevation of alpha1/alpha2 globulin, suggestive of acute inflammation. A punch biopsy was performed on the right breast, and the sample was submitted to the pathology department.

**Fig 3:**
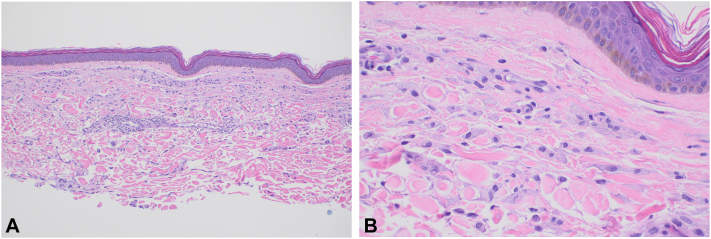
Punch biopsy, hematoxylin-eosin stain showing superficial dermal infiltrate of numerous pale foamy histiocytes. (**A** and **B,** Hematoxylin-eosin stain; original magnification: **A,** ×40; **B,** ×400).

Histopathology revealed an infiltrate of numerous pale foamy histiocytes in the dermis in an interstitial configuration, suggestive of a xanthoma. Repeat biopsy from the contralateral left breast revealed similar histopathology in a nodular conformation (Fig 3). On the basis of her clinical and repeated histologic findings, a diagnosis of DPX was made. As the patient was asymptomatic, no further treatment was pursued. Six months after the initial presentation, the plaques were unchanged in appearance and asymptomatic.

## Discussion

In normolipidemic patients, DPXs have been associated with paraproteinemia (multiple myeloma and monoclonal gammopathy of undetermined significance) and other hematologic malignancies (chronic and acute leukemias and cutaneous lymphomas).^3^^,^^4^ DPX may precede the development of the systemic conditions by several years.^3^ Although the pathogenesis of DPX has not been fully elucidated, widespread planar xathomas involving multiple body sites have been anecdotally associated with the increased incidence of underlying malignancy.^2^^,^^3^ DPX can appear yellow to orange on examination and dermatoscopy; however, these findings may not be appreciated in patients with skin of color, as presented here.^4^ Histopathology of DPX reveals characteristic lipid-laden macrophages, known as foam cells, in clusters or sheets within the dermis. Lymphocytes and Touton giant cells may also be seen. In their series of DPX, Marcoval et al.^3^ observed variable numbers of foam cells with or without the associated histologic features in skin biopsy samples. To our knowledge, here we present the first known case of DPX localized to the bilateral breasts in a patient with skin of color.

The differential diagnosis of DPX includes other infiltrative disorders such as primary cutaneous amyloidosis, pseudoxanthoma elasticum, and lipidized fibrous histiocytosis. Primary cutaneous amyloidosis is often macular or lichenoid with changes in skin pigmentation, and histologically, it demonstrates amyloid deposits in the dermis.^5^ Pseudoxanthoma elasticum is a hereditary disorder involving elastin degeneration with subsequent cutaneous, ocular, vascular, and cardiac lesion formation. The resulting yellow papules and plaques may resemble xanthomas. However, histology reveals calcified and fragmented elastic fibers.^6^ Lipidized fibrous histiocytosis can also present with foam cells on histology; however, further evaluation often reveals associated epidermal hyperplasia, and vascular and sclerotic collagen proliferation in the reticular dermis. Additionally, lipidized fibrous histiocytosis is found to predominantly affect the lower extremities with a solitary, discolored large nodule on clinical examination.^7^

Given that the lesion distribution is limited to the breasts, extragenital lichen sclerosus et atrophicus or Paget disease of the breast should be considered. Extragenital manifestations of lichen sclerosus rarely present without genital involvement (<6% of cases). The associated characteristic ivory, atrophic plaques of lichen sclerosus exhibit epidermal atrophy and upper dermal sclerosis on histopathology, which is not seen with DPX.^8^ Mammary Paget disease is characterized by an eczematoid eruption that involves the nipple and/or areola. Affected patients report pruritus and often have an underlying ductal carcinoma identified on further imaging.^9^

Although a previous series described patients with eye, face, and neck involvement, we demonstrate a case of DPX limited to the bilateral breasts.^3^ Despite their unusual distribution, these plaques were consistent with DPX on skin biopsy of each breast. As seen in our case, the clinical characteristics of DPX may be subtle in patients with skin of color (Fig 1). A diagnosis of DPX should raise concern for an underlying hematologic cause.

The treatment of DPX primarily involves addressing any underlying causes and lipid profile optimization with statin therapy. Even in normolipidemic patients, statin therapy has been found to reduce the size of the xanthoma and decrease the associated cardiovascular risk. Additionally, surgical resection and laser ablation techniques have been attempted with varying success.^10^ Even in the absence of hyperlipidemia and occult malignancy, patients with DPX should be periodically monitored as the clinical appearance of DPX may precede the development of the systemic conditions by several years. Thorough dermatologic evaluation and skin biopsy is crucial to differentiate DPX from other clinically indistinguishable entities.

## Conflicts of interest

None disclosed.
